# {μ-6,6′-Dimeth­oxy-2,2′-[propane-1,3-diylbis(nitrilo­methyl­idyne)]diphenolato}trinitratocopper(II)erbium(III) acetone solvate

**DOI:** 10.1107/S1600536809037787

**Published:** 2009-10-03

**Authors:** Jing-Chun Xing, Yu-Long Bo, Bing Zhang, Wen-Zhi Li

**Affiliations:** a Department of Anesthesiology, The Second Affiliated Hospital, Harbin Medical University, Harbin 150081, People’s Republic of China

## Abstract

In the title complex, [CuEr(C_19_H_20_N_2_O_4_)(NO_3_)_3_]·CH_3_COCH_3_, the Cu^II^ ion is coordinated in a square-planar environment by two O atoms and two N atoms of a Schiff base ligand. The Er^III^ ion is bis-chelated by three nitrate ligands and coordinated by four O atoms of the Schiff base ligand in a slightly distorted bicapped square-anti­prismatic environment.

## Related literature

For a similar copper–lanthanide complex of the same Schiff base ligand as in the title compound, see: Xing *et al.* (2008 ▶). For the isostuctural Sm analog, see: Wang *et al.* (2008 ▶).
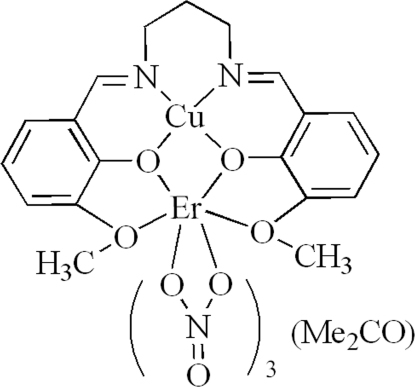


## Experimental

### 

#### Crystal data

[CuEr(C_19_H_20_N_2_O_4_)(NO_3_)_3_]·C_3_H_6_O
*M* *_r_* = 815.28
Triclinic, 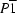

*a* = 9.4142 (19) Å
*b* = 12.151 (2) Å
*c* = 13.439 (3) Å
α = 73.06 (3)°
β = 87.30 (3)°
γ = 72.22 (3)°
*V* = 1398.9 (6) Å^3^
*Z* = 2
Mo *K*α radiation
μ = 3.82 mm^−1^
*T* = 295 K
0.34 × 0.28 × 0.20 mm

#### Data collection

Rigaku R-AXIS RAPID diffractometer
Absorption correction: multi-scan (*ABSCOR*; Higashi, 1995 ▶) *T*_min_ = 0.280, *T*_max_ = 0.460
13866 measured reflections
6335 independent reflections
5654 reflections with *I* > 2σ(*I*)
*R*_int_ = 0.035

#### Refinement

*R*[*F* ^2^ > 2σ(*F* ^2^)] = 0.031
*wR*(*F* ^2^) = 0.094
*S* = 1.12
6335 reflections
388 parameters
H-atom parameters constrained
Δρ_max_ = 1.65 e Å^−3^
Δρ_min_ = −0.99 e Å^−3^

### 

Data collection: *RAPID-AUTO* (Rigaku, 1998 ▶); cell refinement: *RAPID-AUTO*; data reduction: *CrystalStructure* (Rigaku/MSC, 2002 ▶); program(s) used to solve structure: *SHELXS97* (Sheldrick, 2008 ▶); program(s) used to refine structure: *SHELXL97* (Sheldrick, 2008 ▶); molecular graphics: *SHELXTL* (Sheldrick, 2008 ▶); software used to prepare material for publication: *SHELXL97*.

## Supplementary Material

Crystal structure: contains datablocks I, global. DOI: 10.1107/S1600536809037787/lh2899sup1.cif

Structure factors: contains datablocks I. DOI: 10.1107/S1600536809037787/lh2899Isup2.hkl

Additional supplementary materials:  crystallographic information; 3D view; checkCIF report

## Figures and Tables

**Fig. 1.**
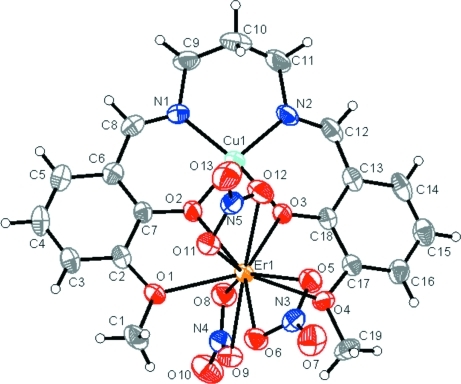
The molecular structure of (I), showing 40% probability displacement ellipsoids. The solvent acetone molecule has been omitted for clarity.

## supplementary crystallographic information

### Comment

The molecular structure of the title complex (I) is shown in Fig. 1. The octadentate Schiff base ligand links the Cu and Er atoms into a dinuclear complex through two phenolate O atoms, which is similar to the coorination in other copper-lanthanide complexes of the same ligand (Xing *et al.*, 2008 and Wang *et al.*, 2008). The Er^III^ ion in (I) is ten-coordinated by four oxygen atoms from the ligand and six oxygen atoms from three nitrate ions. The Cu^II^ center is four-coordinate by two nitrogen atoms and two oxygen atoms from the ligand. The title compound is isostructural with the Sm analog (Wang *et al., *2008).

### Experimental

The title complex was obtained by the treatment of copper(II) acetate monohydrate (0.0499 g, 0.25 mmol) with the Schiff base (0.0855 g, 0.25 mmol) in methanol/acetone (20 ml:5 ml) at room temperature. Then the mixture was refluxed for 3 h after the addition of Erbium (III) nitrate hexahydrate (0.1150 g, 0.25 mmol). The reaction mixture was cooled and filtered; diethyl ether was allowed to diffuse slowly into the solution of the filtrate. Single crystals were obtained after several days. Analysis calculated for C_22_H_26_CuN_5_O_14_Er: C, 32.41; H, 3.21; Cu, 7.79; N, 8.59; Er, 20.52; found: C, 32.40; H, 3.24; Cu, 7.82; N, 8.50; Er, 20.44%.

### Refinement

H atoms bound to C atoms were placed in calculated positions and treated as riding on their parent atoms, with C—H = 0.93 Å (aromatic C), C—H = 0.97 Å (methylene C) and  *U*_iso_(H) = 1.2U_eq_(C) or C—H = 0.96 Å (methyl C) and with *U*_iso_(H) = 1.5U_eq_(C).

### Crystal data

**Table:**

[CuEr(C_19_H_20_N_2_O_4_)(NO_3_)_3_]·C_3_H_6_O	*Z* = 2
*M**_r_* = 815.28	*F*(000) = 804
Triclinic, *P*1	*D*_x_ = 1.936 Mg m^−^^3^
Hall symbol: -P 1	Mo *K*α radiation, λ = 0.71073 Å
*a* = 9.4142 (19) Å	Cell parameters from 12092 reflections
*b* = 12.151 (2) Å	θ = 3.2–27.5°
*c* = 13.439 (3) Å	µ = 3.82 mm^−^^1^
α = 73.06 (3)°	*T* = 295 K
β = 87.30 (3)°	Prism, green
γ = 72.22 (3)°	0.34 × 0.28 × 0.20 mm
*V* = 1398.9 (6) Å^3^	

### Data collection

**Table:**

Rigaku R-AXIS RAPID diffractometer	6335 independent reflections
Radiation source: fine-focus sealed tube	5654 reflections with *I* > 2σ(*I*)
graphite	*R*_int_ = 0.035
Detector resolution: 10.000 pixels mm^-1^	θ_max_ = 27.5°, θ_min_ = 3.2°
ω scans	*h* = −12→12
Absorption correction: multi-scan (*ABSCOR*; Higashi, 1995)	*k* = −15→15
*T*_min_ = 0.280, *T*_max_ = 0.460	*l* = −17→17
13866 measured reflections	

### Refinement

**Table:**

Refinement on *F*^2^	Primary atom site location: structure-invariant direct methods
Least-squares matrix: full	Secondary atom site location: difference Fourier map
*R*[*F*^2^ > 2σ(*F*^2^)] = 0.031	Hydrogen site location: inferred from neighbouring sites
*wR*(*F*^2^) = 0.094	H-atom parameters constrained
*S* = 1.12	*w* = 1/[σ^2^(*F*_o_^2^) + (0.0461*P*)^2^ + 0.9007*P*] where *P* = (*F*_o_^2^ + 2*F*_c_^2^)/3
6335 reflections	(Δ/σ)_max_ = 0.001
388 parameters	Δρ_max_ = 1.65 e Å^−^^3^
0 restraints	Δρ_min_ = −0.99 e Å^−^^3^

### Special details

**Table:**

Geometry. All e.s.d.'s (except the e.s.d. in the dihedral angle between two l.s. planes) are estimated using the full covariance matrix. The cell e.s.d.'s are taken into account individually in the estimation of e.s.d.'s in distances, angles and torsion angles; correlations between e.s.d.'s in cell parameters are only used when they are defined by crystal symmetry. An approximate (isotropic) treatment of cell e.s.d.'s is used for estimating e.s.d.'s involving l.s. planes.
Refinement. Refinement of *F*^2^ against ALL reflections. The weighted *R*-factor *wR* and goodness of fit *S* are based on *F*^2^, conventional *R*-factors *R* are based on *F*, with *F* set to zero for negative *F*^2^. The threshold expression of *F*^2^ > σ(*F*^2^) is used only for calculating *R*-factors(gt) *etc*. and is not relevant to the choice of reflections for refinement. *R*-factors based on *F*^2^ are statistically about twice as large as those based on *F*, and *R*- factors based on ALL data will be even larger.

### Fractional atomic coordinates and isotropic or equivalent isotropic displacement parameters (Å^2^)

**Table:**

	*x*	*y*	*z*	*U*_iso_*/*U*_eq_	
Er1	0.284132 (18)	0.379681 (15)	0.235573 (12)	0.03650 (8)	
Cu1	0.24382 (5)	0.25719 (4)	0.49507 (4)	0.03768 (12)	
O1	0.0400 (3)	0.5379 (3)	0.1950 (2)	0.0443 (7)	
O2	0.1287 (3)	0.3758 (3)	0.3737 (2)	0.0428 (7)	
O3	0.3995 (3)	0.2715 (3)	0.3972 (2)	0.0447 (7)	
O4	0.5566 (3)	0.3518 (3)	0.2453 (2)	0.0455 (7)	
O5	0.4222 (4)	0.2371 (3)	0.1410 (3)	0.0523 (8)	
O6	0.3039 (4)	0.4163 (3)	0.0500 (2)	0.0505 (7)	
O7	0.4131 (5)	0.2827 (4)	−0.0280 (3)	0.0744 (11)	
O8	0.3112 (4)	0.5285 (3)	0.3148 (3)	0.0557 (8)	
O9	0.3219 (4)	0.5766 (3)	0.1492 (3)	0.0505 (7)	
O10	0.3292 (5)	0.7046 (3)	0.2321 (4)	0.0770 (12)	
O11	0.1004 (4)	0.3040 (3)	0.1763 (3)	0.0548 (8)	
O12	0.2438 (4)	0.1704 (3)	0.3018 (3)	0.0619 (9)	
O13	0.0792 (5)	0.1221 (4)	0.2308 (4)	0.0813 (13)	
O14	0.2030 (8)	0.0889 (4)	0.9258 (4)	0.115 (2)	
N1	0.0630 (4)	0.2643 (3)	0.5754 (3)	0.0393 (7)	
N2	0.3884 (4)	0.1296 (3)	0.6007 (3)	0.0492 (9)	
N3	0.3809 (4)	0.3102 (4)	0.0518 (3)	0.0473 (9)	
N4	0.3225 (4)	0.6072 (4)	0.2318 (3)	0.0493 (9)	
N5	0.1395 (5)	0.1959 (4)	0.2355 (3)	0.0530 (10)	
C1	−0.0108 (6)	0.6118 (5)	0.0893 (4)	0.0590 (13)	
H1A	−0.1081	0.6675	0.0901	0.089*	
H1B	−0.0157	0.5608	0.0477	0.089*	
H1C	0.0578	0.6559	0.0604	0.089*	
C2	−0.0648 (4)	0.5340 (4)	0.2695 (3)	0.0384 (8)	
C3	−0.2116 (5)	0.6072 (4)	0.2560 (4)	0.0451 (10)	
H3	−0.2465	0.6643	0.1922	0.054*	
C4	−0.3060 (5)	0.5955 (5)	0.3372 (4)	0.0549 (12)	
H4	−0.4044	0.6455	0.3280	0.066*	
C5	−0.2569 (5)	0.5111 (5)	0.4311 (4)	0.0499 (11)	
H5	−0.3220	0.5039	0.4851	0.060*	
C6	−0.1079 (4)	0.4351 (4)	0.4466 (3)	0.0404 (9)	
C7	−0.0130 (4)	0.4454 (3)	0.3666 (3)	0.0352 (8)	
C8	−0.0651 (5)	0.3414 (4)	0.5451 (3)	0.0429 (9)	
H8	−0.1403	0.3369	0.5921	0.051*	
C9	0.0683 (6)	0.1750 (5)	0.6789 (3)	0.0544 (12)	
H9A	−0.0292	0.1634	0.6914	0.065*	
H9B	0.0911	0.2068	0.7325	0.065*	
C10	0.1825 (7)	0.0557 (4)	0.6868 (4)	0.0637 (15)	
H10A	0.1631	−0.0050	0.7463	0.076*	
H10B	0.1730	0.0323	0.6249	0.076*	
C11	0.3380 (7)	0.0585 (6)	0.6980 (4)	0.0768 (19)	
H11A	0.3436	0.0932	0.7536	0.092*	
H11B	0.4050	−0.0236	0.7176	0.092*	
C12	0.5321 (5)	0.1042 (4)	0.5936 (4)	0.0511 (11)	
H12	0.5889	0.0454	0.6504	0.061*	
C13	0.6146 (5)	0.1543 (4)	0.5107 (3)	0.0425 (9)	
C14	0.7721 (5)	0.1157 (4)	0.5224 (4)	0.0535 (12)	
H14	0.8187	0.0614	0.5846	0.064*	
C15	0.8563 (5)	0.1568 (4)	0.4440 (4)	0.0537 (12)	
H15	0.9598	0.1312	0.4533	0.064*	
C16	0.7885 (5)	0.2370 (4)	0.3499 (4)	0.0471 (10)	
H16	0.8468	0.2649	0.2966	0.057*	
C17	0.6367 (4)	0.2750 (4)	0.3353 (3)	0.0379 (8)	
C18	0.5468 (4)	0.2343 (4)	0.4153 (3)	0.0368 (8)	
C19	0.6431 (5)	0.3926 (5)	0.1567 (4)	0.0560 (12)	
H19A	0.7455	0.3717	0.1796	0.084*	
H19B	0.6036	0.4787	0.1279	0.084*	
H19C	0.6369	0.3543	0.1046	0.084*	
C20	0.3352 (10)	−0.0198 (6)	1.0866 (6)	0.093 (2)	
H20A	0.3551	0.0547	1.0809	0.139*	
H20B	0.4276	−0.0820	1.0879	0.139*	
H20C	0.2851	−0.0422	1.1497	0.139*	
C21	0.2392 (8)	−0.0040 (5)	0.9963 (5)	0.0739 (17)	
C22	0.1955 (10)	−0.1108 (7)	0.9935 (7)	0.106 (3)	
H22A	0.1320	−0.0899	0.9327	0.159*	
H22B	0.1427	−0.1356	1.0547	0.159*	
H22C	0.2834	−0.1758	0.9913	0.159*	

### Atomic displacement parameters (Å^2^)

**Table:**

	*U*^11^	*U*^22^	*U*^33^	*U*^12^	*U*^13^	*U*^23^
Er1	0.03547 (11)	0.03660 (11)	0.02831 (10)	−0.00615 (8)	−0.00097 (7)	−0.00057 (8)
Cu1	0.0400 (2)	0.0358 (2)	0.0294 (2)	−0.0080 (2)	−0.00022 (19)	−0.0010 (2)
O1	0.0421 (15)	0.0431 (16)	0.0349 (14)	−0.0055 (13)	−0.0055 (12)	0.0009 (13)
O2	0.0366 (14)	0.0444 (16)	0.0343 (14)	−0.0014 (12)	0.0014 (12)	−0.0032 (13)
O3	0.0329 (13)	0.0505 (17)	0.0325 (14)	−0.0021 (13)	−0.0021 (11)	0.0044 (13)
O4	0.0379 (14)	0.0522 (17)	0.0390 (15)	−0.0166 (13)	0.0020 (12)	0.0008 (14)
O5	0.0521 (17)	0.0488 (18)	0.0483 (18)	−0.0082 (15)	−0.0009 (15)	−0.0095 (15)
O6	0.0604 (19)	0.0447 (17)	0.0377 (16)	−0.0081 (15)	0.0055 (14)	−0.0077 (14)
O7	0.081 (3)	0.094 (3)	0.053 (2)	−0.018 (2)	0.0095 (19)	−0.039 (2)
O8	0.065 (2)	0.0537 (19)	0.0421 (17)	−0.0119 (17)	0.0031 (15)	−0.0118 (15)
O9	0.0586 (19)	0.0478 (17)	0.0427 (17)	−0.0194 (15)	0.0029 (15)	−0.0062 (14)
O10	0.098 (3)	0.047 (2)	0.090 (3)	−0.028 (2)	−0.012 (2)	−0.018 (2)
O11	0.0509 (18)	0.0488 (18)	0.057 (2)	−0.0146 (15)	0.0011 (15)	−0.0046 (16)
O12	0.070 (2)	0.0414 (18)	0.063 (2)	−0.0108 (17)	−0.0010 (19)	−0.0039 (17)
O13	0.079 (3)	0.067 (2)	0.116 (4)	−0.041 (2)	0.025 (3)	−0.037 (3)
O14	0.172 (6)	0.067 (3)	0.074 (3)	−0.017 (3)	0.004 (3)	0.005 (3)
N1	0.0461 (18)	0.0417 (18)	0.0320 (16)	−0.0179 (16)	0.0033 (14)	−0.0088 (15)
N2	0.059 (2)	0.0365 (18)	0.0332 (18)	0.0025 (17)	0.0028 (17)	−0.0005 (15)
N3	0.0436 (19)	0.058 (2)	0.043 (2)	−0.0201 (18)	0.0057 (16)	−0.0151 (18)
N4	0.047 (2)	0.045 (2)	0.052 (2)	−0.0138 (17)	0.0009 (18)	−0.0081 (18)
N5	0.050 (2)	0.047 (2)	0.062 (2)	−0.0163 (18)	0.0155 (19)	−0.015 (2)
C1	0.063 (3)	0.054 (3)	0.039 (2)	0.000 (2)	−0.014 (2)	0.000 (2)
C2	0.0380 (19)	0.0355 (19)	0.041 (2)	−0.0053 (16)	−0.0058 (17)	−0.0156 (17)
C3	0.039 (2)	0.043 (2)	0.051 (2)	−0.0037 (18)	−0.0100 (19)	−0.017 (2)
C4	0.033 (2)	0.059 (3)	0.071 (3)	0.000 (2)	−0.007 (2)	−0.031 (3)
C5	0.037 (2)	0.060 (3)	0.058 (3)	−0.012 (2)	0.005 (2)	−0.027 (2)
C6	0.040 (2)	0.043 (2)	0.044 (2)	−0.0165 (17)	0.0017 (17)	−0.0168 (18)
C7	0.0326 (17)	0.0333 (18)	0.040 (2)	−0.0084 (15)	−0.0019 (16)	−0.0120 (16)
C8	0.046 (2)	0.051 (2)	0.041 (2)	−0.024 (2)	0.0119 (18)	−0.0196 (19)
C9	0.063 (3)	0.063 (3)	0.034 (2)	−0.027 (2)	0.006 (2)	−0.001 (2)
C10	0.107 (5)	0.042 (2)	0.040 (2)	−0.030 (3)	0.011 (3)	−0.004 (2)
C11	0.081 (4)	0.064 (3)	0.042 (3)	0.009 (3)	0.006 (3)	0.015 (3)
C12	0.059 (3)	0.040 (2)	0.037 (2)	0.006 (2)	−0.011 (2)	−0.0074 (19)
C13	0.043 (2)	0.037 (2)	0.042 (2)	−0.0014 (17)	−0.0072 (18)	−0.0139 (18)
C14	0.048 (2)	0.045 (2)	0.057 (3)	0.004 (2)	−0.024 (2)	−0.016 (2)
C15	0.041 (2)	0.050 (3)	0.072 (3)	−0.011 (2)	−0.010 (2)	−0.021 (2)
C16	0.038 (2)	0.045 (2)	0.062 (3)	−0.0136 (18)	0.000 (2)	−0.020 (2)
C17	0.0371 (19)	0.0336 (19)	0.041 (2)	−0.0082 (16)	−0.0011 (17)	−0.0110 (17)
C18	0.0331 (18)	0.0357 (19)	0.039 (2)	−0.0049 (16)	−0.0026 (16)	−0.0117 (17)
C19	0.050 (3)	0.071 (3)	0.049 (3)	−0.031 (2)	0.012 (2)	−0.009 (2)
C20	0.135 (7)	0.064 (4)	0.082 (5)	−0.034 (4)	0.013 (5)	−0.022 (4)
C21	0.090 (4)	0.056 (3)	0.060 (3)	−0.010 (3)	0.014 (3)	−0.009 (3)
C22	0.116 (6)	0.086 (5)	0.099 (6)	−0.029 (5)	−0.023 (5)	0.001 (4)

### Geometric parameters (Å, °)

**Table:**

Er1—O3	2.305 (3)	C2—C7	1.421 (6)
Er1—O2	2.307 (3)	C3—C4	1.377 (7)
Er1—O6	2.411 (3)	C3—H3	0.9300
Er1—O8	2.428 (4)	C4—C5	1.367 (7)
Er1—O11	2.449 (4)	C4—H4	0.9300
Er1—O5	2.461 (3)	C5—C6	1.410 (6)
Er1—O9	2.462 (3)	C5—H5	0.9300
Er1—O1	2.466 (3)	C6—C7	1.367 (6)
Er1—O4	2.487 (3)	C6—C8	1.451 (6)
Er1—O12	2.577 (4)	C8—H8	0.9300
Er1—N3	2.859 (4)	C9—C10	1.496 (7)
Er1—N4	2.880 (4)	C9—H9A	0.9700
Cu1—O3	1.936 (3)	C9—H9B	0.9700
Cu1—O2	1.938 (3)	C10—C11	1.490 (9)
Cu1—N1	1.966 (4)	C10—H10A	0.9700
Cu1—N2	1.970 (4)	C10—H10B	0.9700
O1—C2	1.372 (5)	C11—H11A	0.9700
O1—C1	1.453 (5)	C11—H11B	0.9700
O2—C7	1.334 (5)	C12—C13	1.424 (7)
O3—C18	1.331 (5)	C12—H12	0.9300
O4—C17	1.379 (5)	C13—C18	1.402 (6)
O4—C19	1.462 (5)	C13—C14	1.413 (6)
O5—N3	1.261 (5)	C14—C15	1.358 (8)
O6—N3	1.265 (5)	C14—H14	0.9300
O7—N3	1.213 (5)	C15—C16	1.391 (7)
O8—N4	1.265 (5)	C15—H15	0.9300
O9—N4	1.270 (5)	C16—C17	1.365 (6)
O10—N4	1.207 (5)	C16—H16	0.9300
O11—N5	1.272 (5)	C17—C18	1.406 (6)
O12—N5	1.261 (6)	C19—H19A	0.9600
O13—N5	1.215 (6)	C19—H19B	0.9600
O14—C21	1.208 (7)	C19—H19C	0.9600
N1—C8	1.277 (6)	C20—C21	1.479 (10)
N1—C9	1.486 (5)	C20—H20A	0.9600
N2—C12	1.298 (6)	C20—H20B	0.9600
N2—C11	1.488 (6)	C20—H20C	0.9600
C1—H1A	0.9600	C21—C22	1.487 (10)
C1—H1B	0.9600	C22—H22A	0.9600
C1—H1C	0.9600	C22—H22B	0.9600
C2—C3	1.384 (5)	C22—H22C	0.9600
			
O3—Er1—O2	64.59 (10)	O7—N3—O6	121.3 (4)
O3—Er1—O6	145.86 (11)	O5—N3—O6	115.7 (4)
O2—Er1—O6	146.93 (11)	O7—N3—Er1	176.1 (3)
O3—Er1—O8	74.16 (12)	O5—N3—Er1	59.1 (2)
O2—Er1—O8	73.18 (12)	O6—N3—Er1	56.8 (2)
O6—Er1—O8	119.26 (12)	O10—N4—O8	121.7 (5)
O3—Er1—O11	116.36 (11)	O10—N4—O9	123.3 (4)
O2—Er1—O11	80.36 (12)	O8—N4—O9	114.9 (4)
O6—Er1—O11	72.98 (12)	O10—N4—Er1	175.8 (4)
O8—Er1—O11	143.46 (12)	O8—N4—Er1	56.7 (2)
O3—Er1—O5	97.20 (11)	O9—N4—Er1	58.3 (2)
O2—Er1—O5	136.85 (11)	O13—N5—O12	121.7 (4)
O6—Er1—O5	52.05 (11)	O13—N5—O11	122.9 (5)
O8—Er1—O5	142.31 (12)	O12—N5—O11	115.4 (4)
O11—Er1—O5	73.57 (12)	O1—C1—H1A	109.5
O3—Er1—O9	118.34 (12)	O1—C1—H1B	109.5
O2—Er1—O9	115.44 (12)	H1A—C1—H1B	109.5
O6—Er1—O9	67.51 (12)	O1—C1—H1C	109.5
O8—Er1—O9	51.80 (11)	H1A—C1—H1C	109.5
O11—Er1—O9	124.51 (11)	H1B—C1—H1C	109.5
O5—Er1—O9	107.65 (11)	O1—C2—C3	125.3 (4)
O3—Er1—O1	127.79 (10)	O1—C2—C7	115.0 (3)
O2—Er1—O1	65.96 (10)	C3—C2—C7	119.7 (4)
O6—Er1—O1	86.32 (11)	C4—C3—C2	119.9 (4)
O8—Er1—O1	76.69 (11)	C4—C3—H3	120.1
O11—Er1—O1	69.64 (11)	C2—C3—H3	120.1
O5—Er1—O1	130.94 (11)	C5—C4—C3	120.8 (4)
O9—Er1—O1	70.46 (11)	C5—C4—H4	119.6
O3—Er1—O4	65.50 (10)	C3—C4—H4	119.6
O2—Er1—O4	125.70 (10)	C4—C5—C6	120.4 (4)
O6—Er1—O4	87.19 (11)	C4—C5—H5	119.8
O8—Er1—O4	74.01 (12)	C6—C5—H5	119.8
O11—Er1—O4	142.51 (12)	C7—C6—C5	119.5 (4)
O5—Er1—O4	69.17 (11)	C7—C6—C8	122.3 (4)
O9—Er1—O4	72.36 (11)	C5—C6—C8	117.9 (4)
O1—Er1—O4	141.89 (11)	O2—C7—C6	123.6 (4)
O3—Er1—O12	67.96 (12)	O2—C7—C2	116.7 (4)
O2—Er1—O12	70.50 (12)	C6—C7—C2	119.7 (4)
O6—Er1—O12	104.96 (12)	N1—C8—C6	128.1 (4)
O8—Er1—O12	135.75 (12)	N1—C8—H8	115.9
O11—Er1—O12	50.34 (12)	C6—C8—H8	115.9
O5—Er1—O12	66.38 (12)	N1—C9—C10	112.3 (4)
O9—Er1—O12	172.45 (11)	N1—C9—H9A	109.1
O1—Er1—O12	109.52 (12)	C10—C9—H9A	109.1
O4—Er1—O12	108.45 (12)	N1—C9—H9B	109.1
O3—Er1—N3	122.43 (11)	C10—C9—H9B	109.1
O2—Er1—N3	149.55 (11)	H9A—C9—H9B	107.9
O6—Er1—N3	26.04 (10)	C11—C10—C9	112.6 (5)
O8—Er1—N3	136.47 (12)	C11—C10—H10A	109.1
O11—Er1—N3	70.11 (11)	C9—C10—H10A	109.1
O5—Er1—N3	26.06 (10)	C11—C10—H10B	109.1
O9—Er1—N3	88.22 (12)	C9—C10—H10B	109.1
O1—Er1—N3	108.59 (11)	H10A—C10—H10B	107.8
O4—Er1—N3	78.13 (11)	N2—C11—C10	112.7 (4)
O12—Er1—N3	84.66 (12)	N2—C11—H11A	109.0
O3—Er1—N4	96.72 (12)	C10—C11—H11A	109.0
O2—Er1—N4	93.97 (12)	N2—C11—H11B	109.0
O6—Er1—N4	93.52 (12)	C10—C11—H11B	109.0
O8—Er1—N4	25.80 (11)	H11A—C11—H11B	107.8
O11—Er1—N4	138.97 (11)	N2—C12—C13	128.7 (4)
O5—Er1—N4	128.06 (12)	N2—C12—H12	115.7
O9—Er1—N4	26.01 (11)	C13—C12—H12	115.7
O1—Er1—N4	71.00 (11)	C18—C13—C14	118.5 (4)
O4—Er1—N4	71.98 (11)	C18—C13—C12	122.8 (4)
O12—Er1—N4	161.53 (13)	C14—C13—C12	118.6 (4)
N3—Er1—N4	113.03 (12)	C15—C14—C13	121.0 (4)
O3—Cu1—O2	79.02 (12)	C15—C14—H14	119.5
O3—Cu1—N1	170.25 (13)	C13—C14—H14	119.5
O2—Cu1—N1	91.24 (14)	C14—C15—C16	120.2 (4)
O3—Cu1—N2	91.25 (14)	C14—C15—H15	119.9
O2—Cu1—N2	169.75 (14)	C16—C15—H15	119.9
N1—Cu1—N2	98.50 (15)	C17—C16—C15	120.4 (5)
O3—Cu1—Er1	40.01 (8)	C17—C16—H16	119.8
O2—Cu1—Er1	40.10 (9)	C15—C16—H16	119.8
N1—Cu1—Er1	130.49 (10)	C16—C17—O4	125.9 (4)
N2—Cu1—Er1	129.72 (11)	C16—C17—C18	120.5 (4)
C2—O1—C1	117.8 (3)	O4—C17—C18	113.5 (3)
C2—O1—Er1	117.6 (2)	O3—C18—C13	122.0 (4)
C1—O1—Er1	122.3 (3)	O3—C18—C17	118.6 (3)
C7—O2—Cu1	128.8 (3)	C13—C18—C17	119.3 (4)
C7—O2—Er1	124.1 (2)	O4—C19—H19A	109.5
Cu1—O2—Er1	107.16 (12)	O4—C19—H19B	109.5
C18—O3—Cu1	129.0 (3)	H19A—C19—H19B	109.5
C18—O3—Er1	123.6 (2)	O4—C19—H19C	109.5
Cu1—O3—Er1	107.31 (12)	H19A—C19—H19C	109.5
C17—O4—C19	116.6 (3)	H19B—C19—H19C	109.5
C17—O4—Er1	117.7 (2)	C21—C20—H20A	109.5
C19—O4—Er1	124.7 (3)	C21—C20—H20B	109.5
N3—O5—Er1	94.9 (3)	H20A—C20—H20B	109.5
N3—O6—Er1	97.2 (2)	C21—C20—H20C	109.5
N4—O8—Er1	97.5 (3)	H20A—C20—H20C	109.5
N4—O9—Er1	95.7 (2)	H20B—C20—H20C	109.5
N5—O11—Er1	100.0 (3)	O14—C21—C20	122.2 (7)
N5—O12—Er1	94.1 (3)	O14—C21—C22	121.1 (7)
C8—N1—C9	115.0 (4)	C20—C21—C22	116.6 (6)
C8—N1—Cu1	124.7 (3)	C21—C22—H22A	109.5
C9—N1—Cu1	120.3 (3)	C21—C22—H22B	109.5
C12—N2—C11	115.1 (4)	H22A—C22—H22B	109.5
C12—N2—Cu1	123.6 (3)	C21—C22—H22C	109.5
C11—N2—Cu1	121.2 (3)	H22A—C22—H22C	109.5
O7—N3—O5	123.1 (4)	H22B—C22—H22C	109.5
			
O2—Er1—Cu1—O3	−162.7 (2)	N4—Er1—O6—N3	140.1 (3)
O6—Er1—Cu1—O3	96.4 (3)	O3—Er1—O8—N4	150.0 (3)
O8—Er1—Cu1—O3	−82.27 (18)	O2—Er1—O8—N4	−142.3 (3)
O11—Er1—Cu1—O3	133.69 (18)	O6—Er1—O8—N4	4.2 (3)
O5—Er1—Cu1—O3	59.59 (18)	O11—Er1—O8—N4	−96.9 (3)
O9—Er1—Cu1—O3	−84.40 (19)	O5—Er1—O8—N4	68.9 (3)
O1—Er1—Cu1—O3	−156.20 (17)	O9—Er1—O8—N4	1.7 (2)
O4—Er1—Cu1—O3	−10.88 (17)	O1—Er1—O8—N4	−73.8 (3)
O12—Er1—Cu1—O3	96.10 (19)	O4—Er1—O8—N4	81.6 (3)
N3—Er1—Cu1—O3	69.6 (2)	O12—Er1—O8—N4	−178.2 (2)
N4—Er1—Cu1—O3	−83.96 (18)	N3—Er1—O8—N4	29.3 (3)
O3—Er1—Cu1—O2	162.7 (2)	O3—Er1—O9—N4	−36.7 (3)
O6—Er1—Cu1—O2	−100.8 (3)	O2—Er1—O9—N4	36.9 (3)
O8—Er1—Cu1—O2	80.46 (17)	O6—Er1—O9—N4	−179.3 (3)
O11—Er1—Cu1—O2	−63.58 (17)	O8—Er1—O9—N4	−1.7 (2)
O5—Er1—Cu1—O2	−137.68 (17)	O11—Er1—O9—N4	132.7 (2)
O9—Er1—Cu1—O2	78.33 (18)	O5—Er1—O9—N4	−145.4 (2)
O1—Er1—Cu1—O2	6.52 (17)	O1—Er1—O9—N4	86.5 (3)
O4—Er1—Cu1—O2	151.84 (17)	O4—Er1—O9—N4	−84.9 (3)
O12—Er1—Cu1—O2	−101.18 (18)	N3—Er1—O9—N4	−163.0 (3)
N3—Er1—Cu1—O2	−127.69 (19)	O3—Er1—O11—N5	−19.5 (3)
N4—Er1—Cu1—O2	78.76 (17)	O2—Er1—O11—N5	−74.7 (3)
O3—Er1—Cu1—N1	176.9 (2)	O6—Er1—O11—N5	125.1 (3)
O2—Er1—Cu1—N1	14.1 (2)	O8—Er1—O11—N5	−118.5 (3)
O6—Er1—Cu1—N1	−86.7 (3)	O5—Er1—O11—N5	70.5 (3)
O8—Er1—Cu1—N1	94.60 (16)	O9—Er1—O11—N5	171.0 (2)
O11—Er1—Cu1—N1	−49.44 (16)	O1—Er1—O11—N5	−142.5 (3)
O5—Er1—Cu1—N1	−123.54 (16)	O4—Er1—O11—N5	64.0 (3)
O9—Er1—Cu1—N1	92.47 (17)	O12—Er1—O11—N5	−2.1 (2)
O1—Er1—Cu1—N1	20.67 (16)	N3—Er1—O11—N5	97.8 (3)
O4—Er1—Cu1—N1	165.99 (16)	N4—Er1—O11—N5	−159.7 (2)
O12—Er1—Cu1—N1	−87.03 (17)	O3—Er1—O12—N5	165.4 (3)
N3—Er1—Cu1—N1	−113.55 (18)	O2—Er1—O12—N5	95.7 (3)
N4—Er1—Cu1—N1	92.91 (16)	O6—Er1—O12—N5	−49.9 (3)
O3—Er1—Cu1—N2	−19.0 (2)	O8—Er1—O12—N5	132.2 (3)
O2—Er1—Cu1—N2	178.3 (2)	O11—Er1—O12—N5	2.1 (2)
O6—Er1—Cu1—N2	77.5 (3)	O5—Er1—O12—N5	−85.7 (3)
O8—Er1—Cu1—N2	−101.25 (19)	O1—Er1—O12—N5	41.5 (3)
O11—Er1—Cu1—N2	114.71 (18)	O4—Er1—O12—N5	−142.0 (3)
O5—Er1—Cu1—N2	40.61 (19)	N3—Er1—O12—N5	−66.4 (3)
O9—Er1—Cu1—N2	−103.38 (19)	N4—Er1—O12—N5	129.8 (4)
O1—Er1—Cu1—N2	−175.18 (18)	O2—Cu1—N1—C8	−9.0 (4)
O4—Er1—Cu1—N2	−29.86 (18)	N2—Cu1—N1—C8	174.2 (4)
O12—Er1—Cu1—N2	77.12 (19)	Er1—Cu1—N1—C8	−18.1 (4)
N3—Er1—Cu1—N2	50.6 (2)	O2—Cu1—N1—C9	170.2 (3)
N4—Er1—Cu1—N2	−102.95 (18)	N2—Cu1—N1—C9	−6.6 (4)
O3—Er1—O1—C2	−26.6 (3)	Er1—Cu1—N1—C9	161.2 (3)
O2—Er1—O1—C2	−6.8 (3)	O3—Cu1—N2—C12	12.7 (4)
O6—Er1—O1—C2	154.7 (3)	O2—Cu1—N2—C12	30.9 (11)
O8—Er1—O1—C2	−84.0 (3)	N1—Cu1—N2—C12	−167.4 (4)
O11—Er1—O1—C2	81.5 (3)	Er1—Cu1—N2—C12	24.7 (5)
O5—Er1—O1—C2	125.4 (3)	O3—Cu1—N2—C11	−170.1 (4)
O9—Er1—O1—C2	−137.9 (3)	O2—Cu1—N2—C11	−151.9 (8)
O4—Er1—O1—C2	−124.6 (3)	N1—Cu1—N2—C11	9.8 (5)
O12—Er1—O1—C2	50.1 (3)	Er1—Cu1—N2—C11	−158.0 (4)
N3—Er1—O1—C2	141.0 (3)	Er1—O5—N3—O7	−176.2 (4)
N4—Er1—O1—C2	−110.3 (3)	Er1—O5—N3—O6	4.6 (4)
O3—Er1—O1—C1	170.8 (3)	Er1—O6—N3—O7	176.1 (4)
O2—Er1—O1—C1	−169.3 (4)	Er1—O6—N3—O5	−4.7 (4)
O6—Er1—O1—C1	−7.8 (3)	O3—Er1—N3—O5	16.1 (3)
O8—Er1—O1—C1	113.4 (4)	O2—Er1—N3—O5	−78.4 (3)
O11—Er1—O1—C1	−81.0 (3)	O6—Er1—N3—O5	175.0 (4)
O5—Er1—O1—C1	−37.2 (4)	O8—Er1—N3—O5	117.6 (3)
O9—Er1—O1—C1	59.6 (3)	O11—Er1—N3—O5	−93.2 (3)
O4—Er1—O1—C1	72.9 (4)	O9—Er1—N3—O5	139.0 (3)
O12—Er1—O1—C1	−112.4 (3)	O1—Er1—N3—O5	−152.4 (2)
N3—Er1—O1—C1	−21.6 (4)	O4—Er1—N3—O5	66.6 (2)
N4—Er1—O1—C1	87.1 (3)	O12—Er1—N3—O5	−43.5 (2)
O3—Cu1—O2—C7	−168.3 (4)	N4—Er1—N3—O5	131.0 (2)
N1—Cu1—O2—C7	11.2 (3)	O3—Er1—N3—O6	−159.0 (2)
N2—Cu1—O2—C7	173.1 (8)	O2—Er1—N3—O6	106.6 (3)
Er1—Cu1—O2—C7	−179.5 (4)	O8—Er1—N3—O6	−57.5 (3)
O3—Cu1—O2—Er1	11.21 (14)	O11—Er1—N3—O6	91.8 (3)
N1—Cu1—O2—Er1	−169.29 (15)	O5—Er1—N3—O6	−175.0 (4)
N2—Cu1—O2—Er1	−7.4 (10)	O9—Er1—N3—O6	−36.1 (3)
O3—Er1—O2—C7	169.3 (3)	O1—Er1—N3—O6	32.6 (3)
O6—Er1—O2—C7	−28.9 (4)	O4—Er1—N3—O6	−108.4 (3)
O8—Er1—O2—C7	89.2 (3)	O12—Er1—N3—O6	141.4 (3)
O11—Er1—O2—C7	−65.3 (3)	N4—Er1—N3—O6	−44.1 (3)
O5—Er1—O2—C7	−118.4 (3)	Er1—O8—N4—O10	175.4 (4)
O9—Er1—O2—C7	58.4 (3)	Er1—O8—N4—O9	−2.8 (4)
O1—Er1—O2—C7	6.6 (3)	Er1—O9—N4—O10	−175.4 (4)
O4—Er1—O2—C7	144.4 (3)	Er1—O9—N4—O8	2.8 (4)
O12—Er1—O2—C7	−116.5 (3)	O3—Er1—N4—O8	−28.9 (3)
N3—Er1—O2—C7	−79.4 (4)	O2—Er1—N4—O8	35.9 (3)
N4—Er1—O2—C7	73.7 (3)	O6—Er1—N4—O8	−176.3 (3)
O3—Er1—O2—Cu1	−10.23 (13)	O11—Er1—N4—O8	115.8 (3)
O6—Er1—O2—Cu1	151.55 (15)	O5—Er1—N4—O8	−133.6 (3)
O8—Er1—O2—Cu1	−90.32 (15)	O9—Er1—N4—O8	−177.0 (4)
O11—Er1—O2—Cu1	115.17 (15)	O1—Er1—N4—O8	98.8 (3)
O5—Er1—O2—Cu1	62.1 (2)	O4—Er1—N4—O8	−90.4 (3)
O9—Er1—O2—Cu1	−121.08 (14)	O12—Er1—N4—O8	3.9 (5)
O1—Er1—O2—Cu1	−172.93 (18)	N3—Er1—N4—O8	−158.5 (3)
O4—Er1—O2—Cu1	−35.2 (2)	O3—Er1—N4—O9	148.1 (2)
O12—Er1—O2—Cu1	63.97 (15)	O2—Er1—N4—O9	−147.1 (2)
N3—Er1—O2—Cu1	101.1 (2)	O6—Er1—N4—O9	0.7 (3)
N4—Er1—O2—Cu1	−105.78 (15)	O8—Er1—N4—O9	177.0 (4)
O2—Cu1—O3—C18	165.2 (4)	O11—Er1—N4—O9	−67.2 (3)
N2—Cu1—O3—C18	−18.1 (4)	O5—Er1—N4—O9	43.4 (3)
Er1—Cu1—O3—C18	176.4 (4)	O1—Er1—N4—O9	−84.2 (3)
O2—Cu1—O3—Er1	−11.24 (14)	O4—Er1—N4—O9	86.6 (3)
N2—Cu1—O3—Er1	165.51 (17)	O12—Er1—N4—O9	−179.1 (3)
O2—Er1—O3—C18	−166.4 (4)	N3—Er1—N4—O9	18.5 (3)
O6—Er1—O3—C18	31.3 (4)	Er1—O12—N5—O13	178.1 (4)
O8—Er1—O3—C18	−87.9 (3)	Er1—O12—N5—O11	−3.5 (4)
O11—Er1—O3—C18	129.8 (3)	Er1—O11—N5—O13	−177.9 (4)
O5—Er1—O3—C18	54.6 (3)	Er1—O11—N5—O12	3.7 (4)
O9—Er1—O3—C18	−59.9 (3)	C1—O1—C2—C3	−9.5 (6)
O1—Er1—O3—C18	−146.3 (3)	Er1—O1—C2—C3	−172.9 (3)
O4—Er1—O3—C18	−8.5 (3)	C1—O1—C2—C7	170.0 (4)
O12—Er1—O3—C18	115.5 (3)	Er1—O1—C2—C7	6.7 (4)
N3—Er1—O3—C18	47.6 (4)	O1—C2—C3—C4	−179.3 (4)
N4—Er1—O3—C18	−75.2 (3)	C7—C2—C3—C4	1.2 (6)
O2—Er1—O3—Cu1	10.24 (13)	C2—C3—C4—C5	−0.7 (7)
O6—Er1—O3—Cu1	−152.05 (16)	C3—C4—C5—C6	0.4 (7)
O8—Er1—O3—Cu1	88.81 (16)	C4—C5—C6—C7	−0.6 (7)
O11—Er1—O3—Cu1	−53.51 (19)	C4—C5—C6—C8	−175.5 (4)
O5—Er1—O3—Cu1	−128.70 (15)	Cu1—O2—C7—C6	−6.5 (6)
O9—Er1—O3—Cu1	116.75 (15)	Er1—O2—C7—C6	174.0 (3)
O1—Er1—O3—Cu1	30.3 (2)	Cu1—O2—C7—C2	173.7 (3)
O4—Er1—O3—Cu1	168.13 (19)	Er1—O2—C7—C2	−5.7 (5)
O12—Er1—O3—Cu1	−67.84 (16)	C5—C6—C7—O2	−178.7 (4)
N3—Er1—O3—Cu1	−135.74 (13)	C8—C6—C7—O2	−4.1 (6)
N4—Er1—O3—Cu1	101.46 (16)	C5—C6—C7—C2	1.0 (6)
O3—Er1—O4—C17	8.3 (3)	C8—C6—C7—C2	175.7 (4)
O2—Er1—O4—C17	33.0 (3)	O1—C2—C7—O2	−1.1 (5)
O6—Er1—O4—C17	−150.6 (3)	C3—C2—C7—O2	178.4 (4)
O8—Er1—O4—C17	87.8 (3)	O1—C2—C7—C6	179.1 (4)
O11—Er1—O4—C17	−93.7 (3)	C3—C2—C7—C6	−1.4 (6)
O5—Er1—O4—C17	−100.4 (3)	C9—N1—C8—C6	−176.5 (4)
O9—Er1—O4—C17	142.1 (3)	Cu1—N1—C8—C6	2.8 (6)
O1—Er1—O4—C17	129.0 (3)	C7—C6—C8—N1	6.0 (7)
O12—Er1—O4—C17	−45.8 (3)	C5—C6—C8—N1	−179.2 (4)
N3—Er1—O4—C17	−126.0 (3)	C8—N1—C9—C10	149.1 (4)
N4—Er1—O4—C17	114.8 (3)	Cu1—N1—C9—C10	−30.2 (6)
O3—Er1—O4—C19	176.5 (4)	N1—C9—C10—C11	74.7 (6)
O2—Er1—O4—C19	−158.7 (3)	C12—N2—C11—C10	−158.5 (5)
O6—Er1—O4—C19	17.6 (4)	Cu1—N2—C11—C10	24.0 (7)
O8—Er1—O4—C19	−104.0 (4)	C9—C10—C11—N2	−70.9 (6)
O11—Er1—O4—C19	74.5 (4)	C11—N2—C12—C13	178.9 (5)
O5—Er1—O4—C19	67.8 (4)	Cu1—N2—C12—C13	−3.7 (7)
O9—Er1—O4—C19	−49.7 (4)	N2—C12—C13—C18	−6.9 (8)
O1—Er1—O4—C19	−62.8 (4)	N2—C12—C13—C14	177.4 (5)
O12—Er1—O4—C19	122.4 (4)	C18—C13—C14—C15	1.4 (7)
N3—Er1—O4—C19	42.2 (4)	C12—C13—C14—C15	177.2 (5)
N4—Er1—O4—C19	−77.0 (4)	C13—C14—C15—C16	−0.8 (7)
O3—Er1—O5—N3	−166.4 (2)	C14—C15—C16—C17	−0.1 (7)
O2—Er1—O5—N3	133.5 (2)	C15—C16—C17—O4	−178.4 (4)
O6—Er1—O5—N3	−2.8 (2)	C15—C16—C17—C18	0.4 (7)
O8—Er1—O5—N3	−93.0 (3)	C19—O4—C17—C16	2.2 (6)
O11—Er1—O5—N3	78.2 (2)	Er1—O4—C17—C16	171.3 (3)
O9—Er1—O5—N3	−43.5 (3)	C19—O4—C17—C18	−176.8 (4)
O1—Er1—O5—N3	35.6 (3)	Er1—O4—C17—C18	−7.6 (4)
O4—Er1—O5—N3	−106.1 (3)	Cu1—O3—C18—C13	13.4 (6)
O12—Er1—O5—N3	131.5 (3)	Er1—O3—C18—C13	−170.7 (3)
N4—Er1—O5—N3	−62.0 (3)	Cu1—O3—C18—C17	−167.8 (3)
O3—Er1—O6—N3	32.7 (4)	Er1—O3—C18—C17	8.1 (5)
O2—Er1—O6—N3	−117.1 (3)	C14—C13—C18—O3	177.7 (4)
O8—Er1—O6—N3	138.3 (2)	C12—C13—C18—O3	2.1 (7)
O11—Er1—O6—N3	−79.4 (3)	C14—C13—C18—C17	−1.1 (6)
O5—Er1—O6—N3	2.8 (2)	C12—C13—C18—C17	−176.7 (4)
O9—Er1—O6—N3	140.4 (3)	C16—C17—C18—O3	−178.6 (4)
O1—Er1—O6—N3	−149.2 (3)	O4—C17—C18—O3	0.4 (5)
O4—Er1—O6—N3	68.4 (3)	C16—C17—C18—C13	0.2 (6)
O12—Er1—O6—N3	−40.0 (3)	O4—C17—C18—C13	179.2 (4)
